# A clinical study on bone defect reconstruction and functional recovery in benign bone tumors of the lower extremity, treated by bone marrow mesenchymal stem cell rapid screening–enrichment–composite system

**DOI:** 10.1186/s12957-021-02198-2

**Published:** 2021-04-05

**Authors:** Lei Wang, Dinghao Luo, Junxiang Wu, Kai Xie, Yu Guo, Yaokai Gan, Wen Wu, Yongqiang Hao

**Affiliations:** 1 Department of Orthopaedics, Shanghai Ninth People’s Hospital, Shanghai JiaoTong University School of Medicine, 639 Zhizaoju Road, Shanghai, 200011 People’s Republic of China; 2 Department of Bone Oncology, Peking University People’s Hospital, Peking University School of Medicine, 11 Xizhimen South Street, Beijing, 100044 People’s Republic of China

**Keywords:** Benign bone tumors of lower extremity, Bone defect reconstruction, Bone marrow mesenchymal stem cell, Rapid screening–enrichment–composite system

## Abstract

**Background:**

With the development of medical technology, credible options for defect reconstructions after the resection of benign bone tumors of the lower extremities have become a high priority. As the current reconstructive methods commonly used in clinical practice have some flaws, new methods of reconstruction need to be explored. We aimed to prepare a new kind of bioactive scaffold for the repair of bone defects through a stem cell rapid screening–enrichment–composite technology system developed by our team. Furthermore, we aimed to investigate the curative effects of these bioactive scaffolds.

**Methods:**

Firstly, cell count, trypan blue exclusion rate, and ALP staining were used to evaluate changes in enrichment efficiency, cell activity, and osteogenic ability before and after enrichment. Then, the scaffolds were placed under the skin of nude mice to verify their osteogenic effects in vivo. Finally, the scaffolds were used for the reconstruction of bone defects after operations for benign bone tumors in a patient’s lower limb. The healing status of the defect site at 1 and 3 months was assessed by X-ray, and the Musculoskeletal Tumor Society (MSTS) score was applied to reflect the recovery of patient limb function.

**Results:**

The system effectively enriched stem cells without affecting the activity and osteogenic abilities of the bone marrow mesenchymal stem cells (BMSCs). Meanwhile, the bioactive scaffolds obtained better osteogenic effects. In patients, the active scaffolds showed better bone integration and healing status, and the patients also obtained higher MSTS scores at 1 and 3 months after surgery.

**Conclusion:**

As a new technique, the rapid screening–enrichment–composite technology of stem cells demonstrates a better therapeutic effect in the reconstruction of bone defects after surgery for benign bone tumors of the lower extremities, which will further improve patient prognosis.

## Background

Bone tumors occur in the bone or its associated tissues with a 0.01% incidence in the population. The incidence ratio among benign bone tumors, malignant bone tumors, and tumor-like lesions is 5:4:1 [1]. Different types of tumors require different treatments due to their different biological characteristics. In terms of benign bone tumors, due to their slow progress, insignificant early symptoms, and a lack of sufficient attention, the lesions are often found after having caused extensive bone destruction and carry a higher risk of pathological fractures or joint function damage.

As benign bone tumors usually do not pose a threat to patient’s lives, the demands for improved postoperative limb function are often higher than those for malignant bone tumors. There is an increasing demand relating to how doctors can provide the most reliable reconstruction of these bone defects after complete removal of the tumor in order to ensure the maximum function of the affected limb.

Confirmation of surgical protocol is mainly based on the evaluation of the biological behaviors of the benign bone tumor using the Enneking surgical staging system. Local curettage or bone cement filling and marginal resection are the most commonly used surgical methods and are often combined with physical, chemical, and drug inactivation measures during the operation to further reduce recurrence rates and improve recovery.

However, the reconstruction of bone defects after excision still presents difficulties in the treatment of benign bone tumors and has also become a hotspot for domestic and foreign scholars. At present, many bone implant materials are used in clinical practice, among which autologous bone transplantation and allograft bone transplantation are the most effective methods [2]. Due to the destruction of the donor bone area and an insufficient bone mass, autogenous bone transplantation has limited use in the reconstruction of bone defects after huge benign bone tumor resections [3–5]. Allografts, by contrast, have the advantage of being more widely sourced and of being used in largely unrestricted quantities. Therefore, it is the preferred way to reconstruct larger bone defects after resection of the bone tumor. However, if the local osteogenic capacity of the defect is impaired, the slow rate of bone penetration and bone integration increases the risk of rejection, absorption, and nonunion of the allograft. Therefore, how to bio-modify an existing allogenic bone material to promote its osteogenic ability is a key area in further improving the efficacy of benign bone tumor resections and in reducing postoperative complications.

In 2007, the “Diamond Concept” theory was proposed by Giannoudi [6]. The theory indicated that four factors involving growth factor, bone conduction stents, BMSCs, and mechanical stability play leading roles during the bone repair process. Based on this theory, any material used to repair bone defects should cover as many of the above factors as possible. This multi-factor combination therapy, providing mechanical stability and appropriate biological stimulation, can achieve better results than a monotherapy [7, 8]. However, how to obtain a large number of active BMSCs in vitro in order to construct a bioactive bone defect repair scaffold is still a relatively difficult problem. At present, most clinical applications of BMSCs still need to be amplified in vitro to obtain sufficient quantities. Currently, adequate cell count remains the primary concern in the clinical application of BMSCs, and in vitro amplification culture is still the main method. However, this requires a long preoperative preparation time, which increases the cost and contamination probabilities. Besides, prolonged in vitro division and proliferation of BMSCs can lead to a decreased expression of the stem cell characteristic genes, chromatin variation [9, 10], and even malignant tumor formation [11].

In view of this, a bone marrow stem cell rapid screening–enrichment–composite system has been independently developed by our research group. This system utilizes the high adhesion characteristics of bone marrow mesenchymal stem cells (BMSCs) to rapidly screen and enrich stem cells by circulating bone marrow blood in a one-time, completely closed and fast filtration pipeline. This means that BMSCs can be rapidly adhered to the surface and the inside of porous bone defect filling materials. In other words, porous bone defect filling materials, such as beta-TCP, can be used as a carrier for BMSCs, to gather a large number of the BMSCs in a defect after the resection of a benign bone tumor, so as to improve the efficacy of the consequent bone defect repair. This study retrospectively analyzed the relevant data of patients with lower extremity benign bone tumors treated in our department from July 2013 to April 2016, in order to further clarify the effects of this technique in the bone defect restoration of benign bone tumors and to provide a good theoretical basis for its clinical application.

## Methods

### Patient inclusion criteria and exclusion criteria

Inclusion criteria: (1) benign bone tumors occurring in the long bones of the limbs with local medullary cavity involvement associated with large bone defects after surgery and with bone grafting indications suitable for rapid stem cell screening and enrichment technology; (2) no hematopoietic systemic disease; and (3) a follow-up time of no less than 12 months and with complete follow-up data available.

Exclusion criteria: (1) defects involving all of the medullary cavity requiring a large segment osteotomy; (2) pathological findings showing that the tumor had abundant cells with a high atypia degree, indicating a high possibility of recurrence; and (3) a loss of visitor.

### Case data

A total of 22 patients were included in this study and divided into two groups: an experimental and control group. In the experimental group, the 11 patients using the enriched stem cell technique for bone defect repair included 8 males and 3 female, with an average age of 40.6 years old. The pathological diagnoses were as follows: 7 cases of giant cell tumors, 2 cases of fatty sclerosing mucinous fibrous tumor, and 2 cases of fibrous dysplasia. In the control group, conventional methods were carried out to repair the bone defect. There were 11 cases in total, including 7 males and 4 females. The average age was 30 years old. Their pathological diagnoses were 6 cases of giant cell tumor, 4 cases of non-ossifying fibroma, and 1 case of atypical cartilaginous tumor, respectively. This study was carried out after the approval of the Shanghai Ninth People’s Hospital Ethics Committee. We have obtained the consent for publication from the patient.

### Preparation of the beta-TCP enriched stem cells

After general anesthesia, the anterior superior iliac spine was routinely disinfected and a towel was laid. A total of 75–80 mL of bone marrow blood was extracted. Following which, porous beta-TCP particles (Shanghai Bio-lu Biomaterials, Shanghai, China) with around a 3–5 mm diameter and 75% ± 10% average porosity were put into a filter box, and the stem cell rapid screening–enrichment–composite system was then assembled. A total of 65–70 mL of bone marrow blood was injected into the system and filtered through the porous beta-TCP particles at a frequency of 70 Hz for 10 min. Finally, the bioactive beta-TCP particles were prepared.

### Nucleated cell count, cell viability, and osteogenesis evaluation

For each sample, 1 mL of pre- and post-enrichment bone marrow was treated with a red blood cell lysis buffer (BioTime, Shanghai, China). Then, the nucleated cells (NCs) were counted using a hemocytometer (Beckman Coulter, Brea, California, USA). Cell viability was assessed by the trypan blue exclusion rate (Vi-CELL XR Cell Viability Analyzer Software, Beckman Coulter). The difference between the pre- and post-enrichment bone marrow was estimated for each patient. Osteogenic abilities were also evaluated by the ratio of integrated option density (IOD) and related cell area (IOD/area).

### Osteogenesis evaluation of porous beta-TCP loaded with BMSCs

A small amount of porous beta-TCP particles was implanted subcutaneously into 3-month-old nude mice in order to evaluate in vivo osteogenesis effects. The control group was treated with equal-quality pure TCP particles. In detail, a transverse incision of about 0.5 cm long was made on both sides of the spine. Porous beta-TCP particles enriched with BMSCs were placed subcutaneously on the left, while the control groups were placed on the right. Three weeks later, the nudes were sacrificed, and the materials were taken out and stained with picric acid and magenta to observe the inside osteogenesis under microscope. Osteogenic effect was evaluated by the ratio of integrated option density (IOD) and related cell area (IOD/Area).

### Intraoperative operating

After general anesthesia, we firstly spent 10 min collecting the bone marrow blood. Then, bone tumor resection combined with bone grafting was performed after the diseased limbs had been disinfected. During the operation, all bone tumor focus areas were completely removed and the tumor boundaries were inactivated with anhydrous ethanol. In the test group, about 25–47 mL of porous beta-TCP particles enriched with BMSCs were implanted into the defect area, while in the control group, about 18–30 mL of pure beta-TCP particles were implanted into the defect area.

### Evaluation of postoperative efficacy

X-ray films at 1 week after surgery were used as the baseline. Bone formation at 3 months after surgery was taken as the main observation index to evaluate the degree of bone healing in the bone defect site for both groups. At the same time, Musculoskeletal Tumor Society (MSTS) scores for the two groups were recorded and the functional recoveries of the affected limbs were compared. The degree of bone defect healing was evaluated by two orthopedic surgeons and one radiologist. Simultaneously, the MSTS score was assessed and recorded through a “single blind” method by two orthopedic surgeons who did not participate in this project.

### Statistical method

SPSS Statistics 20.0 (IBM, America) was used for the statistical analysis. The mean, standard error of the mean and *P* values based on two-tailed *t* tests were calculated. Differences were considered significant at *P* < 0.05.

## Results

### Detection of enrichment efficiency, cell viability, and osteogenic ability

**Fig. 1:**
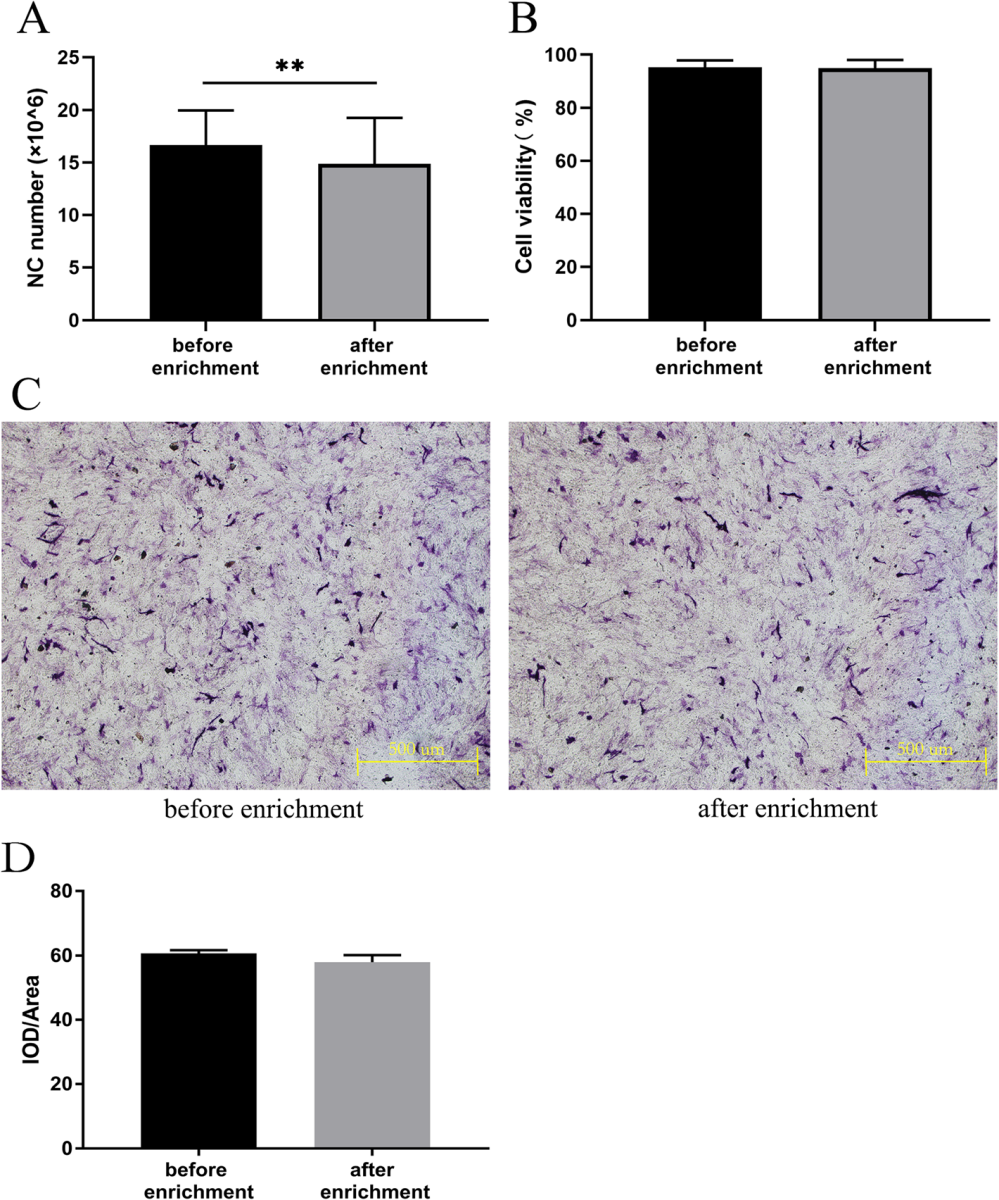
The number of nucleated cells (NC), cell viability, and osteogenic ability before and after enrichment of bone marrow blood (*n* = 11). **a** The number of NC after enrichment was statistically lower than that in the original bone marrow blood (*P* < 0.01). **b** There was no difference in cell viability before and after enrichment (*P* > 0.05). **c** No significant changes in the osteogenic abilities were observed before and after enrichment. **d** There was no significant difference in osteogenic abilities before and after enrichment (*P* > 0.05)

The number of nucleated cells in the blood of the bone marrow, both before and after enrichment, was measured for each patient, and it was found that the number of cells after enrichment (14.89 ± 4.37 × 10^6^) were significantly less than the number before enrichment (16.67 ± 3.29 × 10^6^). Wilcoxon’s signed rank testing confirmed that this showed a significant statistical difference (Fig. 1a). The mean cell viability of the bone marrow NCs was evaluated with a trypan blue exclusion rate. Our results indicated that there was no difference between cell viability before and after enrichment (95.29% ± 2.59% vs. 94.97% ± 3.06%). When the cells collected before and after enrichment (Fig. 1b) proliferated in the right number, ALP staining was performed according to the standard steps after osteogenesis had been induced for 7 days. It was proven that the enrichment process did not affect the osteogenic properties of BMSCs (Fig. 1c, d). In summary, the stem cell rapid screening–enrichment–composite system effectively enriched stem cells without affecting their vitality and osteogenic abilities.

### Evaluation of the osteogenic effects of MSC/TCP bioactive scaffolds in vivo

**Fig. 2:**
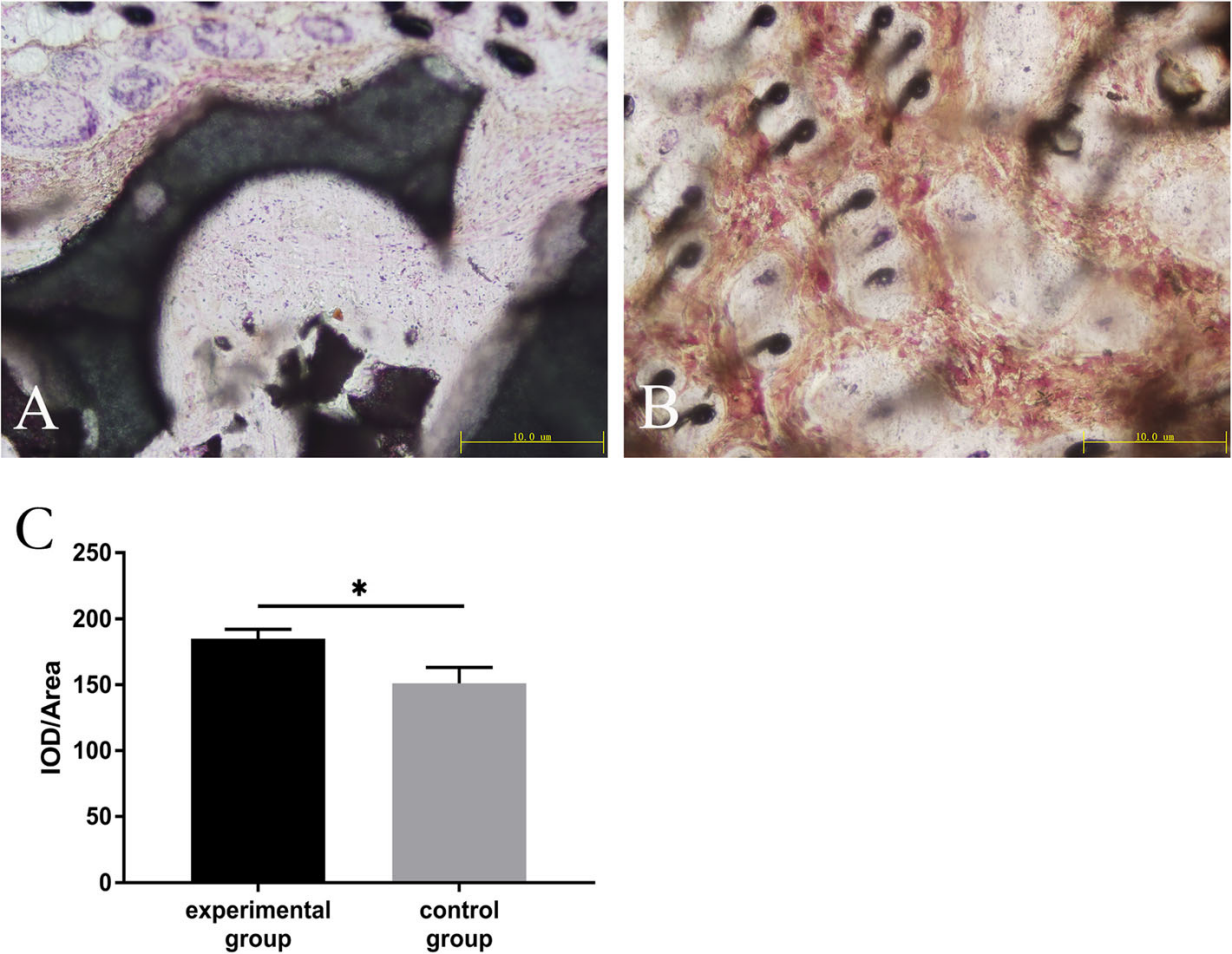
Evaluation of the osteogenic effects of mesenchymal stem cell (MSC)/TCP bioactive scaffolds in vivo (*n* = 5). **a** The control group showed less osteogenesis. **b** However, there were more new bone components in the experimental group, which distributed in a reticular manner. **c** The osteogenic effects in the experimental group were statistically higher than the control group (*P* < 0.05)

MSC/TCP bioactive scaffolds were removed from the subcutaneous tissues of nude mice at 3 weeks after implantation. After hard tissue embedding, sectioning, and staining, the results showed that the MSC/TCP bioactive scaffolds induced a higher degree of osteogenesis when compared to the control group (Fig. 2).

### X-ray features

**Fig. 3:**
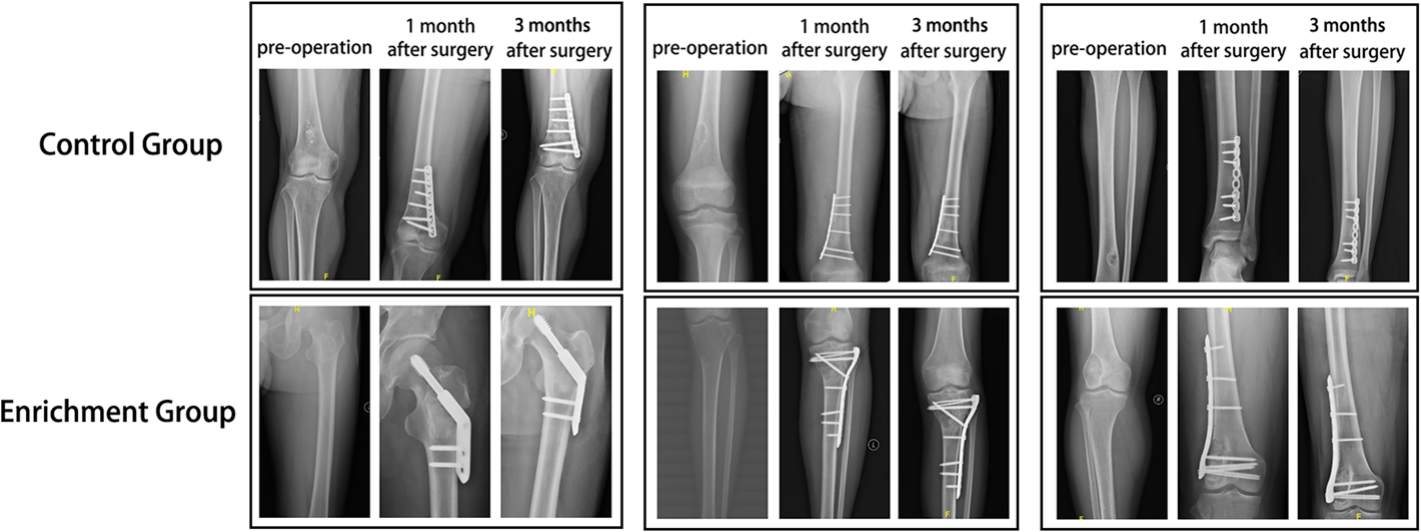
X-ray findings for the MSC/TCP bioactive scaffolds in repairing bone defects after benign bone tumor resection. The upper row showed three patients in the experimental group, while three patients in the control group are placed in the lower row

One month after the operation, X-ray findings showed that the tumors in both the experimental and control groups had been completely removed with no obvious recurrences. The material filled into the bone defects had not been completely absorbed. However, the experimental group had a better osteogenic effect at 1 month after surgery, showing a higher density in the defect area. At 3 months after the operation, the filling material was further absorbed, which was again better in the experimental group as compared to the control group. The experimental group also showed significant improvements at 1 month in terms of bone healing, while the control group had no significant progress in the 3 months after surgery (Fig. 3). Therefore, our results confirm that MSC/TCP bioactive scaffold materials prepared by a stem cell rapid screening–enrichment–composite system had better bone healing effects in repairing bone defects after benign bone tumor resection.

### Functional evaluation of the affected limb

We recorded MSTS scores for the two groups in order to evaluate the patient’s lower limb function. It was found that the patients were experiencing obvious pain and limited movement during weight-bearing activities and almost all of the preoperative MSTS scores were of a very poor grade. When the tumor was completely removed and the defect was reconstructed, the MSTS scores of the patients were improved at 1 month after the operation in both groups. However, the MSTS scores of the experimental group were higher than those of the control group due to the better bone healing. The same results were also achieved in the MSTS scores at 3 months postoperatively (Table 1). These results confirm that bone defects repaired by MSC/TCP bioactive scaffold materials prepared through a stem cell rapid screening–enrichment–composite system can lead to a better limb function for affected patients.

**Table 1:** Preoperative and postoperative MSTS scores of patients. The preoperative Musculoskeletal Society (MSTS) scores for the two groups were almost all below a “poor grade.” MSTS scores for the two groups were significantly improved at 1 and 3 months after the operation, and the scores of the experimental group were higher than those of the control group at the same time points

	Experiment group	Control group	*P* value
Pre-operation	**10.82 ± 1.601**	**10.91 ± 1.814**	**0.902**
1 m after operation	**20.91 ± 1.868**	**17.95 ± 2.911**	**0.004**
3 m after operation	**26.00 ± 1.673**	**22.91 ± 2.256**	**0.002**

## Discussion

How to obtain a large number of active BMSCs in vitro is a relatively difficult problem. To solve this problem, some early studies used gradient centrifugation to obtain a marrow nucleated cell suspension containing abundant mesenchymal stem cells, which could reach a concentration of 2579 ± 1121 osteoprogenitor cells/mL [12]. Similar techniques were also used to obtain cell suspensions rich in mesenchymal stem cells in the early stages of our study, and the BMSC concentration of per unit volume was increased by about 4.3 times [13]. Although centrifuge technology can obtain BMSCs with a high concentration, it cannot be separated from expensive cell sorters, which greatly increases the treatment cost for patients and is difficult to promote. To this end, we further improved and developed the rapid screening–enrichment–composite system of bone marrow stem cells. The system mainly utilizes the strong adhesion characteristics of stem cells to make the BMSCs adhere directly to the interior of the scaffold material through perfusion, avoiding the loss of liquid active components. The complete set of equipment needed for the composite system are disposable products and the pipelines are of a sealed design, which avoids the risk of bacterial contamination. Secondly, bone marrow blood collection can be completed within the 15 min before surgery. BMSC enrichment and its combination with a scaffold material can be carried out simultaneously with the tumor resection, thus decreasing patient waiting time before and during surgery. Thirdly, during in the whole process of the composite system, no exogenous reagents are needed, which increases the safety of the enriched stem cells. Therefore, this system has a high working efficiency and good clinical feasibility.

In this study, we applied the bioactive scaffold materials prepared by the stem cell rapid screening–enrichment–composite system to reconstruct bone defects after the removal of benign bone tumors in the lower limbs. All of these patients had good bone healing at 3 months postoperatively. The functional recovery of the lower limbs was evaluated with the MSTS scoring system, which indicated that these patients had a better, statistically significant functional status at 3 months after surgery when compared to the control group. In addition, the analysis of blood cultures, cell numbers and cell vitality before and after enrichment suggested that (1) this system effectively isolated microbial contamination through strict aseptic operation and a completely closed-loop design, resulting in negative blood culture rates of 100% and improved safety; (2) the system had a high enrichment efficiency of stem cells—after a short time filtrating porous materials, the number of BMSCs in bone marrow blood could decrease by 83.6%, while the number of nucleated cells, red blood cells, and platelets had no significant changes; and (3) compared with pure beta-TCP, the bioactive scaffold prepared with this system had better osteogenic abilities in vivo. Therefore, active bone grafting materials prepared through a rapid screening–enrichment–composite technique can effectively treat residual bone defects after the resection of benign bone tumors, which is not only conducive to the rapid recovery limb function, but can also be used as a more effective alternative therapy for autologous bone transplantation.

## Conclusion

In summary, as a new technique for the treatment of bone defects, the rapid screening–enrichment–composite technology of stem cells can avoid many disadvantages associated with autologous bone grafting and implements a new autologous bone repair approach for the defected area. However, the small sample size and limited follow-up times of this study is its main deficiency, meaning large randomized controlled clinical trials need to be carried out to further confirm these results, and this is our intention for future research.

## Data Availability

The datasets used and analyzed during the current study are available from the corresponding author on reasonable request.
